# Does Epileptiform Activity Represent a Failure of Neuromodulation to Control Central Pattern Generator-Like Neocortical Behavior?

**DOI:** 10.3389/fncir.2017.00078

**Published:** 2017-10-18

**Authors:** Roger D. Traub, Miles A. Whittington, Stephen P. Hall

**Affiliations:** ^1^Department of Physical Sciences, IBM Thomas J. Watson Research Center, New York City, NY, United States; ^2^Department of Biology, Hull York Medical School, University of York, York, United Kingdom

**Keywords:** delta oscillation, spike-wave epilepsy, NPY interneuron, VIP interneuron, neocortex, disinhibition

## Abstract

Rhythmic motor patterns in invertebrates are often driven by specialized “central pattern generators” (CPGs), containing small numbers of neurons, which are likely to be “identifiable” in one individual compared with another. The dynamics of any particular CPG lies under the control of modulatory substances, amines, or peptides, entering the CPG from outside it, or released by internal constituent neurons; consequently, a particular CPG can generate a given rhythm at different frequencies and amplitudes, and perhaps even generate a repertoire of distinctive patterns. The mechanisms exploited by neuromodulators in this respect are manifold: Intrinsic conductances (e.g., calcium, potassium channels), conductance state of postsynaptic receptors, degree of plasticity, and magnitude and kinetics of transmitter release can all be affected. The CPG concept has been generalized to vertebrate motor pattern generating circuits (e.g., for locomotion), which may contain large numbers of neurons – a construct that is sensible, if there is enough redundancy: that is, the *large* number of neurons consists of only a *small* number of classes, and the cells within any one class act stereotypically. Here we suggest that CPG and modulator ideas may also help to understand cortical oscillations, normal ones, and particularly transition to epileptiform pathology. Furthermore, in the case illustrated, the mechanism of the transition appears to be an exaggerated form of a normal modulatory action used to influence sensory processing.

## Introduction

It is perhaps odd that the study of invertebrate CPGs has been so influential, conceptually, in the field of neocortical physiology (Yuste et al., 2005). After all, most CPGs have only a few neurons; the cortex has many millions. One reason for the influence of CPGs may be this: their detailed characterization proved that one could deconstruct a neuronal network into its constituent pieces – the neurons and the chemical and electrical synapses – and study these pieces alone and in pairs – and thereby account for the collective properties of the whole network (e.g., Traub et al., 2005). It turned out, however, that a “neuronal network,” even a CPG, is really an abstraction: no neuronal network (in biology) exists as a well-defined circuit with precisely fixed parameters, because membrane and synaptic properties are constantly in flux owing, at least in part, to neuromodulation-dependent, continuous turnover of membrane proteins (Carlier et al., 2006). Even so, the notion of such a deconstruction into constituent pieces proved immensely seductive. Perhaps one could apply an analogous intellectual framework to building a copy of the whole cortex, or even whole brain, by a similar – albeit much more laborious – approach?

Without wishing to engage in polemics, we argue in this review that the CPG-analogous intellectual framework does make sense in a restricted context: for example, in the case of cortical oscillations and sz, when there is a high degree of redundancy – physicists might call it “degeneracy,” although the word sounds odd in a biological context – in the behavior of neuronal subtypes.

To make our case, we describe an example of an *in vitro* cortical oscillation, induced by a modulator (an ACh agonist) and modulator-antagonist (of dopamine D1 receptors), that corresponds to a normal oscillation observed *in vivo* during deep sleep, and to a lesser extent in awake cortex; and we shall describe how perturbations involving additional modulators [serotonin, ACh acting via nicotinic receptors, VIP, and neuropeptide Y (NPY)] convert the normal oscillation into an abnormal oscillation resembling SpW sz EEG patterns (Tucker et al., 2009; Van Bogaert, 2013).

## Basic Properties of Invertebrate CPGS

The lives of mobile animals depend on stereotyped (more-or-less) movement patterns – even in humans. Thus, one has breathing, chewing, walking, or swimming as examples of (relatively) rhythmic patterns; and yawning and swallowing as non-rhythmic patterns. Invertebrates having, for the most part, relatively small nervous systems, may devote quite small numbers (sometimes dozens, or even less) of neurons for the generation of such patterns (Selverston, 2010). Examples include (but certainly are not limited to) swimming in *Tritonia* (Getting, 1983), the stomatogastric ganglion of lobsters and crabs (Marder and Bucher, 2007; Katz, 2016), and the circuitry governing contractions in the leech circulatory system (Calabrese et al., 2016).

In some instances, such as the stomatogastric ganglion, the CPG can be physically isolated from the rest of the animal (analogous to the preparation of a mammalian *in vitro* brain slice) for study. One may then record from single neurons in isolation to characterize intrinsic membrane properties (with the caveat that the cellular anatomy of single neurons may be extremely complex, with intracellular access only possible at the soma and not in small “neurites”); and one can, at least in principle, record from every pair of neurons to characterize each chemical and electrical synapse. One may, as well, bathe the preparation in modulatory substances (e.g., octopamine), stimulate, or inhibit single cells during patterned activity to examine the consequences, manipulate the membrane properties of individual cells via “dynamic clamp,” and likewise physically ablate single cells. There is the additional advantage that many of the neurons are identifiable (Kandel et al., 1967) from individual to individual, so that experiments from day to day, or year to year should be reproducible [although detailed membrane properties of any one identified cell will, in general, vary (Selverston, 2010)].

Thus, the study of invertebrate CPGs allows the realization, at least partially, of a neurophysiologist’s dream – to account for the operations of a neural circuit, in terms of the physical properties of the constituent neurons and of the pairwise synaptic (chemical and electrical) interactions.

However, the existence of modulator effects complicates matters. There are many modulators, and each one can alter membrane and synaptic properties, sometimes in ways that are known (at least partly), other times in unknown ways. Through the action of modulators, a given circuit can generate one or another behavioral pattern (Nusbaum and Beenhakker, 2002); and an individual neuron can participate in different circuits (Marder, 2012). The very concept of a CPG becomes muddied, as the CPG is not, in fact, completely defined by its cell membrane and its synapses, but only by those objects as exist in the presence of various chemicals, the latter changing over time. This same idea applies to vertebrate brains (Harris-Warrick, 2010).

### Extension of the CPG Idea to Vertebrate Locomotion Circuits, and Circuits Producing Other Rhythmic Motor Patterns

**FIGURE 1:**
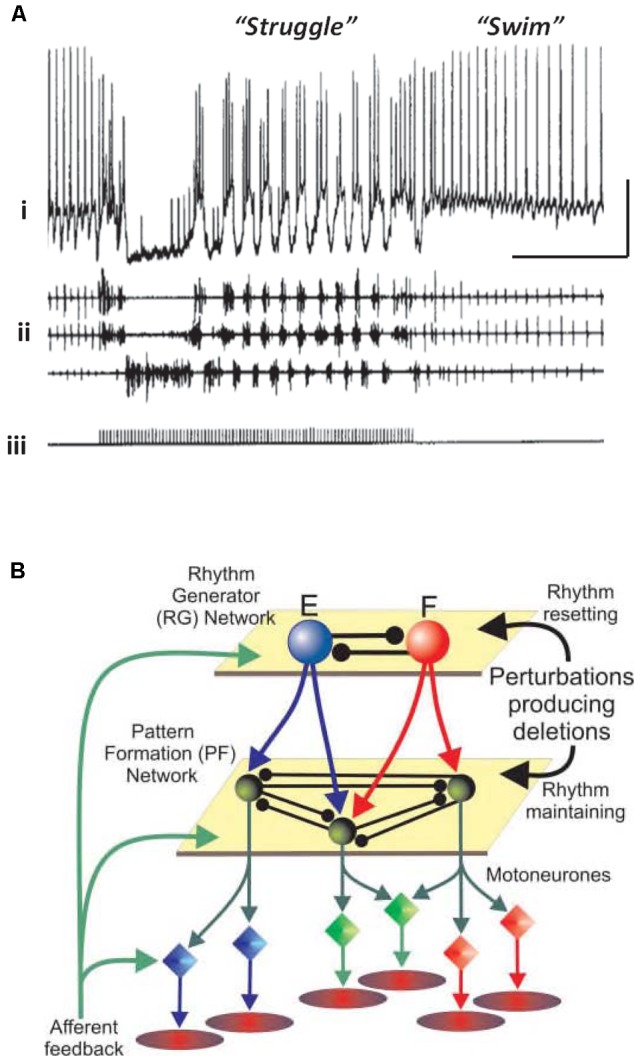
Simple central pattern generators (CPGs) generate multiple output dynamics. **(A)** Two distinct network patterns in the spinal cord of a paralyzed *Xenopus laevis* embryo. The “struggle” pattern was evoked by repetitive electrical stimulation of the skin. The recording of the presumed motorneuron was intracellular, and was extracellular for the motor roots. From Soffe (1993) with permission. Scale bars, 20 mV, 1 s. **(B)** A proposed model of locomotor pattern generation in mammalian spinal cord. In this model, one set of neurons (RG, or rhythm generator, top) produces an underlying population oscillation. Signals are fed to an intermediate network that uses the oscillatory signal to produce one, or another, spatiotemporal pattern (PF, or pattern formation network, middle). PF signals are fed, in turn, to the motorneurons which drive the muscles. In this scheme, a given relatively fixed rhythm can lead to a number of different patterns downstream. From Rybak et al. (2006) with permission.

Vertebrates also have neuronal circuits that generate relatively stereotyped, but modifiable, rhythmic motor behaviors – swimming, walking, and the like. The CPG notions, and terminology, have been extended to such vertebrate circuits, but there are important differences from the invertebrate case. Vertebrate circuits contain more neurons than in the invertebrate case, the neurons are not often identifiable (although often *classifiable*, as in the case of motorneurons), it is not technically possible to isolate the vertebrate CPG, and it can be difficult to be sure which randomly picked neurons actually belong to the CPG, or influence it. On the other hand, vertebrate CPGs can be at least partially isolated – in an isolated spinal cord preparation, for example – and CPG outputs can be monitored quantitatively, either electrically (recording from ventral roots, for “fictive swimming”) or optically. Rhythmic behavior in the vertebrate case can be induced and modified by bath application of modulatory substances (such as serotonin and NMDA), and it is possible to switch from one pattern to another pattern by stimulation (**Figure 1A**). In the illustrated example, the stimulation was electrical, but it is possible that the stimulation induces transmitter release with activation of metabotropic receptors, by analogy with the effects of electrical stimulation in the hippocampal slice (see Whittington et al., 1997a,b). In addition, abrupt changes from burst to more tonic firing are seen in STG for dopamine application alone (Marder and Thirumalai, 2002; Bucher et al., 2003).

There has been extensive computational modeling of vertebrate CPGs. A noteworthy example is the lamprey spinal cord swimming generator (Grillner, 2006; Buschges et al., 2011). Factors that help to make such a modeling effort feasible include these: the ability to divide the preparation into smaller, but functionally and anatomically meaningful, modules (left vs. right, spinal cord segments); and the ability to divide the neuronal population into a relatively small number of subtypes. The results of experimental and modeling efforts in this field have been impressive indeed.

One principle that has been suggested for a vertebrate spinal cord locomotor CPG (**Figure 1B**) is this: that the CPG has a hierarchical organization (Rybak et al., 2006), one level generating a basic rhythm, an intermediate “pattern forming” level operating on and restructuring the basic rhythm (and also providing feedback to the primary oscillator), and finally an “execution” level of motorneurons that drives the actual muscle fibers – certain fibers at certain times, and not others. This type of hierarchical organization was suggested by the phase of oscillations that would occur after a missed beat (Rybak et al., 2006). We suggest that a similar principle applies to at least some types of cortical oscillation, with the pattern forming operation possibly being critical for cortical function.

## A Comparison of CPG Behavior to Modulator-Dependent Cortical Oscillations

As is the case for invertebrate nervous systems, and phylogenetically older portions of vertebrate nervous systems, in cortical structures the neurons can be classified into a finite number of types – by location of somata and dendrites, axonal branching patterns, transmitters and co-transmitters released, chemical markers, receptors expressed, and so forth. [It is true that controversy exists concerning just how the classification ought to be carried out (Battaglia et al., 2013)]. It is likewise the case that, during *in vitro* collective behaviors, induced by bath application of modulatory substances/receptor activators (kainate, carbachol, etc.), defined cell types tend to behave in defined ways (e.g., Klausberger et al., 2003, 2005; Roopun et al., 2006) – a concept that is hard to quantify owing to precedents for altered electrophysiological behaviors depending on neuromodulatory state, but nevertheless useful, and indeed essential for modeling purposes.

Hence, the CPG framework may be applicable to thinking about cortical oscillations, at least overt, stable examples modeled *in vitro*. That is not to imply that the framework instantly illuminates all of the biological questions; but rather that certain principles that have applied to CPGs, however, diverse CPGs may be (Selverston, 2010), may also apply to the cortex. This is no more apparent than when considering the effects of neuromodulation. For example, we saw above how stimulus or neuromodulation with dopamine may transform a CPG output from rhythmic bursting to near-continuous firing (Soffe, 1993; Bucher et al., 2003). A remarkably similar change in output pattern can be seen in neocortex under varying modulation by dopamine (**Figure 3**). This transition was attributed to dopamine’s ability to potentiate ectopic action potentials generated at the mid-axon level in the Bucher et al.’s (2003) study. While this effect was mediated via enhancement of a slow-inward current (Ih) in the axon (Ballo et al., 2010), we do not yet know the mechanism for this transition in cortex. It is interesting to speculate though that a similar process may be occurring. Dopamine has also been shown to enhance both Ih in layer 5 (L5) pyramidal neurons (Rosenkranz and Johnston, 2006) and ectopic axonal action potential generation in mossy fibers in rat (Vivar et al., 2012).

### How (Many) Cortical Oscillations Differ from Classical CPGs

**FIGURE 2:**
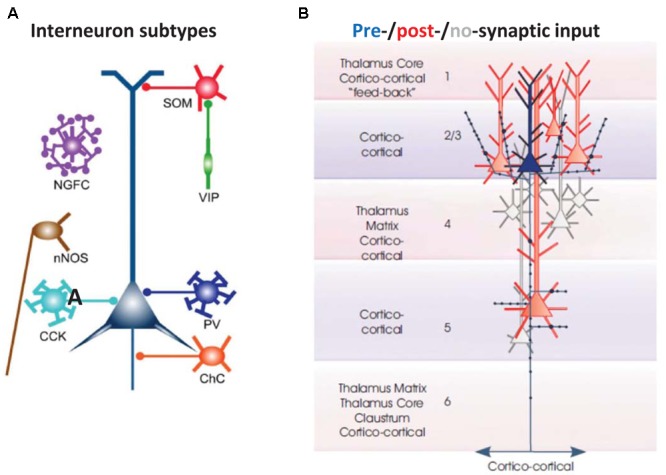
Basic cellular components of a local neocortical circuit. **(A)** Some of the interneuronal circuitry in neocortex, indicated highly schematically. The figure shows axoaxonic chandelier cells (ChC, orange); parvalbumin-positive basket cells (PV, blue); CCK basket cells (light blue); neuronal nitric oxide synthase cells (nNOS, brown), neurogliaform cells (NGFC, purple); dendrite-contacting somatostatin cells (SOM, red); and predominantly interneuron-contacting VIP cells (green). Most of the connections between interneurons, and gap junction connections, are not shown. The computer model (Traub et al., 2005; Carracedo et al., 2013) contains chandelier cells, basket cells, NGFC, and SOM cells (and, in a more recent version, VIP cells). From Taniguchi (2014) with permission. **(B)** Excitatory postsynaptic connectivity of L2/L3 neocortical pyramidal cells. They synapse on each other, and on L5 pyramidal cells: both those with large tufts in L1 (these tending to lie in more superficial L5), and also those with smaller, shorter apical dendrites (these tending to lie deeper in L5). In addition, L2/L3 pyramids form longer range cortico-cortical connections. From Thomson and Lamy (2007) with permission.

**FIGURE 3:**
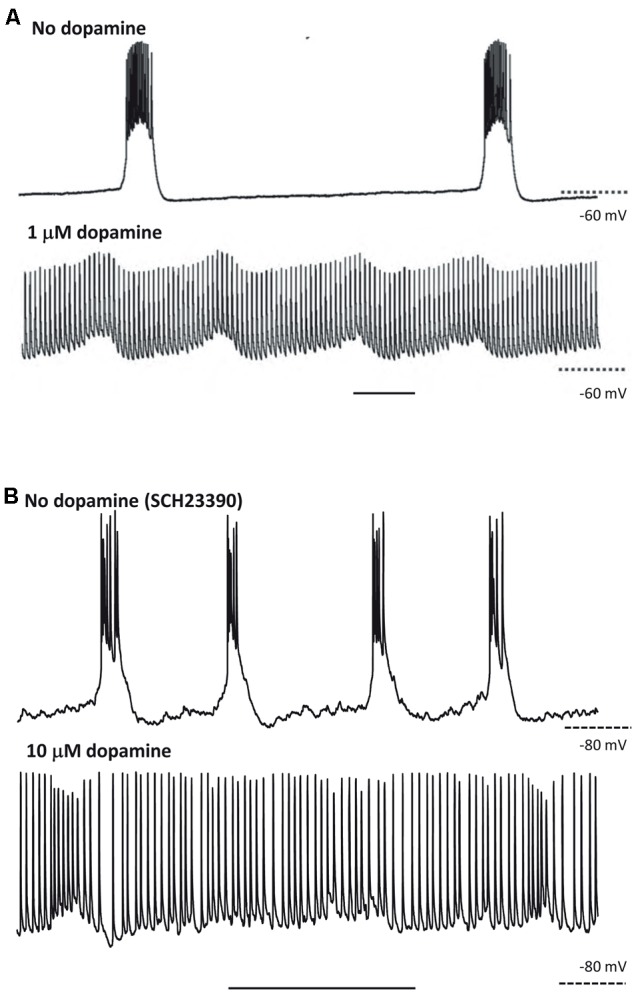
At least some responses to neuromodulators are shared between CPGs and neocortex. **(A)** CPG output recorded in a stomatogastric motorneuron shows exquisite sensitivity to dopaminergic neuromodulation. Note the near-collapse of the slow, rhythmic burst discharges and the emergence of near-tonic action potential discharges. From Bucher et al. (2003) with permission. Scale bar, 1 s. **(B)** Output of L5 IB neurons in rat parietal neocortex. Rhythmic bursting occurs persistently in the presence of the non-hydrolysable ACh analog carbachol (4 mM) and low dopaminergic tone (SCH23390, 10 mM). Wash-out of the dopaminergic antagonist SCH23390, followed by application of 10 mM dopamine depolarises these neurons, almost abolished bursting and promotes trains of single spikes (Hall and Whittington, unpublished). Scale bar, 1 s.

Despite the many crude functional and anatomical similarities that can be seen (e.g., **Figures 1B, 2, 3**), there are important differences between cortical oscillations and CPGs that should not be underemphasized. Two differences stand out. *First*, classical CPGs produce a physical, observable motor output. Even in situations where motor output itself is not visible (if the animal is paralyzed, or the CPG has been isolated from most or all of the rest of the animal), “fictive” output will be observable by appropriate electrical or optical recordings. Such a feature allows an intellectually satisfying synthesis of neural activity with concrete behavior. In contrast, most cortical oscillations, and none *in vitro*, have a motor correlate, so that the physical significance of the oscillation is generally unclear, and subject to speculation and controversy (Singer, 1999). *Second*, as alluded to above, the number of neurons engaged in cortical oscillations is large (particularly *in vivo*); and while partial classification of neuronal subtypes is possible, a complete and precise classification appears to be beyond reach. We might mention as well that, *in vivo*, cortical oscillations do not exist in isolation: because of the extensive interconnections of any one cortical region with other cortical regions, with the thalamus, the basal ganglia, olfactory bulbs, and so forth. This is a situation that we must live with.

To simplify matters, let us consider just a few possible functions of cortical oscillations that may be defined independently of a fictive output: (a) acting as a clock (rhythm-generator), that establishes relative phases for action potentials (e.g., O’Keefe and Recce, 1993); (b) allowing “cell assemblies” to be formed, that is collections of cells firing “together” (pattern formation), i.e., at similar phases with respect to an underlying oscillation (Leznik et al., 2002; Kopell et al., 2011). These notions are, we believe, compatible with the functions of cells participating in a multi-layered CPG (**Figure 1B**), yet are more general and more abstract.

## Modulator-Dependent Transformation of Sleep-Related Cortical Oscillations Into Epileptiform Activity

Electroencephalography and ECoG signals during slow-wave sleep (i.e., non-REM or non-rapid-eye-movement sleep) contain a rich variety of interrelated waves at 4 Hz or less. *First*, there is the slow oscillation of sleep, described by Steriade et al. (1993a,b), originally observed in anesthetized animals but also present without anesthesia; this typically occurs at <1 Hz, and is associated with large, alternating, depolarizations and hyperpolarizations (“up- and down-states”), synchronized among most cortical neurons. At least in entorhinal cortex *in vitro*, this type of rhythm is controlled in part by ATP-gated K^+^ channels (Cunningham et al., 2006). *Second*, and the subject of our discussion, are so-called delta waves at ∼1–4 Hz. As we shall see, the associated large depolarizations in delta waves occur in some, but not other, cortical neurons. Interestingly, delta waves also occur in the waking state, as well as in sleep; but during the waking state, they are of smaller spectral power, more localized and transient than in sleep – perhaps reflecting a reduced number of neurons recruited into the rhythm and altered long-range functional connectivity. Waking-associated delta may play an important role in information processing (Lakatos et al., 2005, 2008; Sachdev et al., 2015).

**FIGURE 4:**
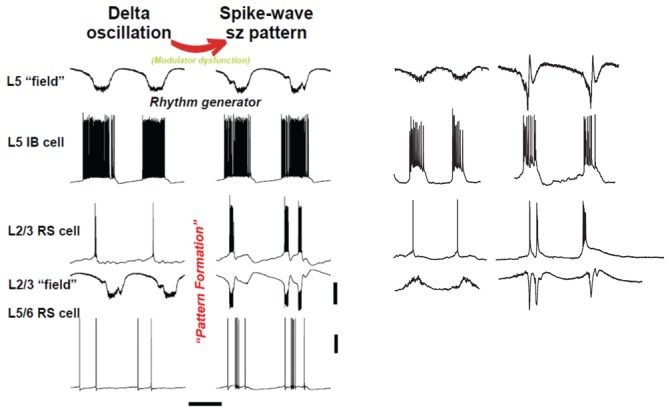
“Rhythm” and “pattern” generators in neocortex are critically dependent on neuromodulation. Left panel shows computer model (described briefly in text) suggests an interpretation similar to **Figure 1**: we propose that the subnetwork of L5 IB cells, interconnected with one another by synaptic [and probably electrical (Wang et al., 2010) connections], acts as a delta frequency “rhythm generator,” with period determined in part by GABA_B_ receptor-mediated inhibition (not shown in this figure). The output of the L5 IB subnetwork is fed, by a multiplicity of connections, to the “pattern generator,” consisting of RS cells in both superficial and deep layers, along with a variety of interneurons (not shown). The transition from delta to spike-wave (SpW) is induced by changing levels of neuromodulators (principally NPY and VIP), whose primary effects are in synaptic transmission in superficial layers (see text); however, the pattern of deep RS cells also switches on going from sparse, single spiking to multiple spikes and bursts – a result of altered activities in superficial layers. Scale bars (model): 20 mV (averages manifest as field potentials and individual cells), 200 ms. Right panel shows corresponding experimental data in normal, delta rhythm conditions, and when superficial layer peptidergic neuromodulation is disrupted. Data from Carracedo et al. (2013); Hall et al. (2015), and unpublished. Experimental traces were not recorded simultaneously. Note this manipulation to the neuromodulatory influences acting on the circuit disrupts the “pattern formation” behavior of RS cells while leaving the rhythm generation in IB cells almost completely untouched – in stark contrast to the effects of dopamine modulation (**Figure 3**). Scale bars, 0.2 mV (fields), 20 mV (cells), 500 ms (delta), and 800 ms (SpW).

We have been able to develop (Carracedo et al., 2013; **Figure 4**) a computational and *in vitro* neocortical slice model of delta waves in rat secondary somatosensory/parietal cortex. The platform for the model is detailed in Traub et al. (2005) and upon it we have manipulated receptor-mediated signals for two modulatory substances: ACh and dopamine. This was motivated by a number of precedents: The delta rhythm not only occurs during sleep, where it is associated with memory consolidation in a neuromodulator-dependent manner (Feld and Born, 2017), but also during wakefulness where it is involved in decision-making (Nácher et al., 2013); many sz subtypes occur preferentially during slow-wave sleep (Park et al., 1998); spike and wave type epilepsies tend to occur during drowsiness and, when precipitated directly by sleep, can be severe (Japaridze et al., 2016); some spike and wave-like epilepsies are related to genetic mutations leading to altered cholinergic and dopaminergic neuromodulatory state (Arvaniti et al., 2016).

We used a low concentration (2–4 μM) of carbachol to activate muscarinic and nicotinic ACh receptors, and 10 μM SCH23390 (Ongini et al., 1987; Bourne, 2001) to block dopamine D1 receptors. Both ACh (Sarter and Bruno, 1997) and dopamine (Nieoullon, 2002; Boulougouris and Tsaltas, 2008) are important in arousal and attention and powerfully modulate CPG outputs via their widespread distribution and multiplicity of actions (see above). Cholinergic inputs to the cortex derive largely from basal forebrain (Buzsáki and Gage, 1989; Kalmbach et al., 2012), dopaminergic inputs largely from the midbrain ventral tegmental area (Walsh and Han, 2014).

In order to make sense of the physiology, a few points about cortical circuitry and about our assumptions are worth setting out:

(1) Cortical interneurons (**Figure 2A**) mostly belong to one of three large classes, within which there are subdivisions (Tremblay et al., 2016): parvalbumin (PV), SOM, and 5HT3a, the latter named after 5HT receptor (Thomson and Lamy, 2007; Vucurovic et al., 2010; Gentet, 2012; Taniguchi, 2014; Tremblay et al., 2016). Importantly, there is cross-reactivity between 5HT3a and nicotinic ACh receptors (Yan et al., 2006). PV interneurons include “classical” basket cells and most axoaxonic interneurons. SOM interneurons largely make their synaptic contacts onto apical dendrites of pyramidal neurons. A subtype of 5HT3a interneurons consists of VIP interneurons, which (among other actions) inhibit other interneurons, especially SOM ones – an interaction with experimentally demonstrable consequences for local network excitability (Pfeffer et al., 2013; Fu et al., 2014). Finally, NPY interneurons can be of 5HT3a or SOM types. Our data suggest that VIP interneurons inhibit NPY ones (Hall et al., 2015), in addition to other sorts of SOM dendrite-contacting interneurons. At least onto L5 pyramidal neurons, NPY effects include diminution of evoked excitatory synaptic currents, and increases in evoked inhibitory synaptic currents (Bacci et al., 2002). Thus, expected actions of activating VIP interneurons would be increasing cortical excitability, via disinhibition of pyramidal cell dendrites.
(2) Secondary somatosensory/parietal cortex has limited L4. Thus, a reasonable detailed understanding of local circuit function in this region does not require a detailed reconstruction of the complex inter- and intralaminar neuronal connectivity profiles (and indeed multiple neuronal subtypes) additionally influencing activity in primary sensory regions.
(3) There are fewer connectivity studies in the secondary somatosensory/parietal cortical region, using simultaneously recorded principal neurons, as compared with other cortical regions, such as barrel cortex. We shall assume, based on other studies (Thomson and Lamy, 2007; Lefort et al., 2009), that principal neuron subpopulations are connected within and between each other, with descending connections from L2/L3 being especially prominent, and with at least some degree of upward connectivity from deep layers to superficial ones (**Figure 2B**).
(4) In our experimental analysis, we group together principal cells by the location of their somata and their intrinsic firing properties (RS vs. IB), concentrating on L2/L3 RS cells, and “deep” (mostly L5) RS and IB cells. There is, of course, much more diversity in the actual tissue (e.g., in L6, or with respect to IB cells in superficial layers) than this tentative classification would imply. Interneurons were also identified by somatic location and intrinsic firing properties; and, on occasion, by reconstruction of filled cells combined with staining characteristics [for NPY or VIP (Hall et al., 2015)].

The upper rows of **Figure 4** illustrate the CPG-like phenomenology of principal cell behavior during this model, purely neocortical (i.e., no thalamic participation) delta: there are large fields in both superficial and deep layers, which are tightly time-locked to highly regular bursts of action potentials (riding on depolarizing envelopes) in L5 IB cells. The subnetwork of L5 IB cells appears to behave as a primary rhythm generator (c.f. **Figure 1B**), because of the stereotypic behavior of these cells, even after perturbation of the system by some, but not all (**Figure 3**) modulators. In other words, the synergistic combination of intrinsic properties of IB cells and their near-invariant large synaptic inputs serves to provide a robust, invariant rhythm on the rest of connected neocortex. Thus, if specific computations, in terms of output patterns dependent critically on spatiotemporal input patterns, are being performed by the deep IB cells it is hard to see what such computations consist of. In addition, deep IB cells do not project over long distances within neocortex, instead providing afferent inputs exclusively to subcortical structures (Kim et al., 2015). It is worth noting that the details of the intrinsic properties and connectivity profiles of these IB neurons differ from those commonly found in invertebrate rhythm generator neurons, but general similarities do exist. These latter cells function usually via low threshold voltage-gated conductances generating rebound spiking from mutual inhibition (**Figure 1B**). IB neurons generate rebound spiking via intermediate-threshold calcium conductances on the rebound from GABA_B_ receptor-mediated inhibition “shared” among local IB cell populations via local circuit interneurons (Carracedo et al., 2013).

In contrast RS cell outputs are highly variable. They may fire single spikes, runs of spikes, or bursts in a manner related to both the dominant intrinsic conductances activated and their collective synaptic inputs – both factors being sensitive to peptidergic neuromodulatory state. Variability in spike timing is a critical feature determining information content in neuronal networks: The greater the variability the more complex the patterns that can be generated to represent sensory information (Fetterhoff et al., 2015). If the spike variability of RS neurons is curtailed by the emergence of rhythmic bursting (as for IB cells, above) by pathological shifts in neuromodulatory state, then the library of patterns, and thus information content, able to be generated collapses.

Once delta oscillations have been induced, modulator “tone” can be further altered by block of the nicotinic component of cholinergic neuromodulation (dTC 10 μM), so that the remaining “external” modulator actions in the tissue are entirely via muscarinic receptor activation. This alteration robustly has two major effects (Hall et al., 2015), which we believe to be causally related. The *first* effect is on the superficial and deep fields, and on the firing patterns of superficial and deep RS principal cells (**Figure 4**, ‘SpW sz pattern’): there is a large “spike” in the fields (a terminology derived from the EEG literature – “SpW”). The “spike” is temporally correlated with burst firing in the RS cells, or sometimes continuous rhythmic firing in deep RS cells (**Figure 6**). Interestingly delta rhythm generation continues in the deep layers, much as before, although at a lower frequency in the experimental situation.

**FIGURE 5:**
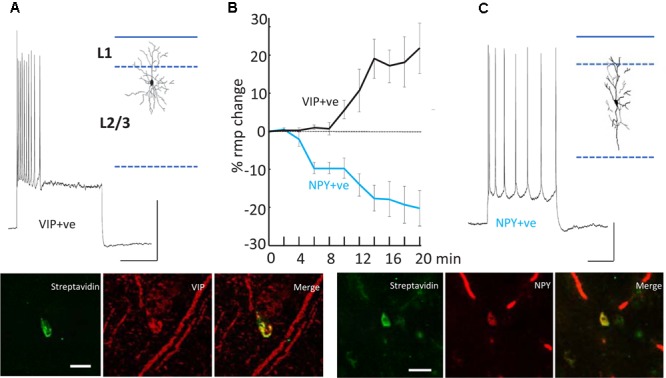
Differential modulation of VIP+ and NPY+ interneuron excitability. **(A)** Example recording from a VIP immunopositive (VIP+ve) neuron in superficial layers of parietal cortex. Response to current injection (0.5 nA) is shown and the somatodendritic cytoarchitecture is shown as inset. Scale bars, 20 mV, 50 ms. Below the streptavidin signal from the recorded cell and the VIP-immunoreactivity of the same cortical region are shown. Scale bar, 20 mM. **(B)** Mean (±SEM) resting membrane potential changes preceding SpW generation following bath application of TC (time = 0). Tonic hyperpolarization of NPY-immunopositive, interneurons (blue line, *n* = 5) preceded tonic depolarization of VIP-immunopositive, interneurons (black line, *n* = 5). **(C)** Example recording from an NPY immunopositive (NPY+ve) neuron in superficial layers of parietal cortex. Response to current injection (0.2 nA) is shown and the somatodendritic cytoarchitecture is shown as inset. Scale bars, 20 mV, 50 ms. Below the streptavidin signal from the recorded cell and the NPY-immunoreactivity of the same cortical region are shown. Scale bar, 20 mM. Data from Hall et al. (2015) with permission.

The *second* effect of dTC on delta was to alter the behavior of VIP and NPY interneurons, concurrently with the appearance of SpW – specifically, exciting the activity of VIP interneurons and suppressing the activity of NPY interneurons (**Figure 5**). The net effect is to reduce the spike output from NPY interneurons and recruit previously silent VIP interneurons into the underlying delta rhythm (Hall et al., 2015). Both of these effects would be expected, in view of known electrophysiology (enhanced glutamate release, diminished GABA release), to enhance the excitability within superficial cortical layers; and indeed, in our network model, the transition from delta to SpW was achieved by augmenting the conductance of excitatory synapses between superficial pyramids, together with diminishing synaptic inhibition (onto pyramids) in superficial layers. Furthermore, combining dTC with either a blocker of VIP receptors, or of NPY receptors, demonstrated certain effects that would be expected according to our hypothesis concerning VIP interneuron-induced disinhibition: for example, blocking VIP receptors delayed the onset of SpW produced by dTC; while blocking NPY receptors accelerated it (Hall et al., 2015).

**FIGURE 6:**
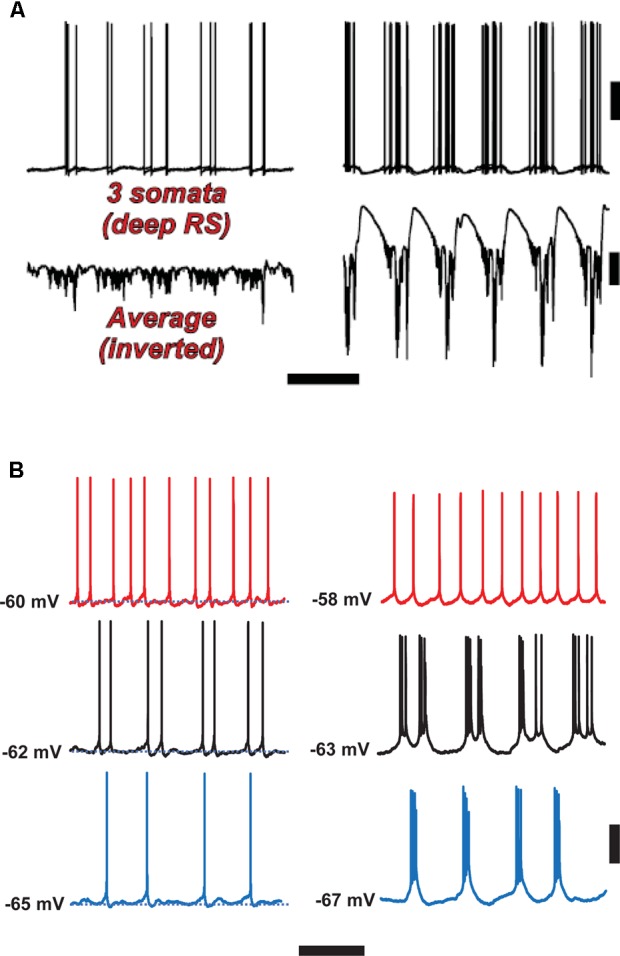
Spike-wave discharges merge rhythm generation and pattern generation components of the delta rhythm. Computer model **(A)** suggests that the firing of deep RS cells is more temporally dispersed during delta, as compared with SpW. Temporal dispersion would allow specific time of deep RS action potentials to convey information. Normal delta rhythm is shown in the right column, SpW activity in the left. Scale bars, 20 mV (cells), 2 mV (averages), 500 ms. **(B)** In experiments, the temporal pattern of deep RS cell firing is dependent on membrane potential (as is the case in the model, see Carracedo et al., 2013); both in the model **(A)** and in the experiment **(B)**, bursting is more likely in the SpW state (Right) than in the delta state (left). From Carracedo et al. (2013) (**B**, Left) with permission, and unpublished data. Scale bars, 20 mV, 600 ms.

Note that in both experiment and model (**Figures 4, 6**), firing of *deep* RS cells is also enhanced by SpW conditions. We attribute this, primarily, to the strong synaptic excitation of deep RS cells by superficial RS cells, which start bursting during SpW. That conclusion, however, is not easy to demonstrate directly in the slice.

An overview of the delta/SpW paradigm, suggested by the functional CPG structure (**Figure 1B**), the similarities between this and the layers of cortex (**Figure 2B**), and combined with our data, is that (as noted above) delta is the *primary rhythm generator*, produced by interconnected L5 IB cells, and by recurrent GABA_B_ receptor-mediated inhibition [not discussed here – see the original papers (Carracedo et al., 2013; Hall et al., 2015)]; while superficial layers, and deep RS cells (along with interconnected interneurons) constitute the *pattern formation network*. This viewpoint could be relevant, as it is the pattern formation network that contains the neurons forming the majority of cortico-cortical interconnections.

Not surprisingly then, the above two peptidergic neuromodulators – VIP and NPY – have been shown to play critical roles in a number of cognitive processes: VIP [via potent effects on inhibitory balance in neocortex (Batista-Brito et al., 2017)] has been shown to affect memory formation and recall (Chaudhury et al., 2008); NPY enhances cognitive performance in behavioral rodent models and alters fear-conditioning via excitability changes in prefrontal cortex (Vollmer et al., 2016). The normal balance between rhythm and pattern generation in this model – with rhythm generation taking the form of the delta oscillation and pattern generation the sparse, theta-like activity in superficial layers – is a common feature of cortical activity involved in sensory selection (Schroeder and Lakatos, 2009). Disruption of this process may therefore underlie, at least in part, the “absences” that typify spike- and wave-related epilepsies.

## Clinical Correlations

Electroencephalography (intracranial or scalp) and MEG SpW patterns can be recorded in a number of clinical contexts, some of which are as follows:

(1) Single SpW complexes, or short runs, can occur focally (Perruca et al., 2014).
(2) Generalized ∼3 Hz SpW occurs with classical absence, wherein it tends to have abrupt onset and offset, arising out of a normal EEG background (Porter, 1993). Pierre Gloor et al. have proposed a relation between this type of activity and thalamocortical sleep spindles (Kostopoulos et al., 1982), a complex subject into which we do not enter here.
(3) Spike-wave mixed with polyspike-wave at ∼4–6 Hz occurs as a generalized interictal EEG pattern in juvenile myoclonic epilepsy (Dreifuss, 1989).
(4) “Atypical” SpW, slower than 3 Hz and of gradual onset and offset, occurs as a generalized EEG pattern in a number of childhood epileptic encephalopathies; it may persist for hours during sleep (CSWS or continuous SpW of sleep); such syndromes include Lennox-Gastaut and Landau-Kleffner epileptic aphasia (Nickels and Wirrell, 2008; Sánchez Fernández et al., 2012; Van Bogaert, 2013; Issa, 2014; Auvin et al., 2016).

A further possible link between clinical epilepsy and our experimental findings lies in the observations, based on molecular genetics in human families with epilepsy, of a relation between nicotinic receptors and epilepsy (Sutor and Zolles, 2001), although the epilepsies in question here are of focal onset and tend to arise in the frontal lobes – so that we are hesitant to draw conclusions from our data relative to familial nocturnal frontal lobe epilepsy.

It is therefore not straightforward to relate our findings to a specific type of epilepsy, especially given the difficulty in obtaining human tissue, suitable for intracellular recordings, from patients with generalized epilepsies, i.e., patients who are not candidates for focal neocortical resections. There may, however, be possibilities for exploring the experimental effects of modulators, such as we have discussed, in human tissue from patients with focal epilepsies.

### An Hypothesis on Why Absence Seizures Are Associated With “Absence,” But Not Falling Down

A remarkable clinical feature of a classical absence attack is that the patient becomes disconnected from the environment, yet does not fall to the ground. [There may be subtle twitches, myoclonic jerks, or eye blinking (Joshi and Patrick, 2007).] Both the cognitive disconnection, and the preservation of posture, require explanation. With respect to the latter issue, posture, the cortical control of posture in humans appears to be complicated and not at all understood (Mori et al., 1995), to such an extent that it is hard to develop a sensible hypothesis.

With respect to the cognitive disconnection, however, we suggest the following. As **Figure 2B** [from Thomson and Lamy, 2007] indicates, excitatory cortical/cortical connections are largely made by L2/L3 pyramids and smaller L5 pyramids, putatively RS cells. As mentioned above, large L5 pyramids, often IB, do have local excitatory connections, but send long-range axons to subcortical structures, e.g., corpus striatum, thalamus, superior colliculus, pontine nuclei, spinal cord, etc. – see also Hattox and Nelson (2007). Experimentally (**Figure 6B**), we note that the firing of deep RS cells, during delta, is much less stereotyped than is the firing of deep IB cells. We propose that the relative timing of action potentials amongst the deep RS cells carries important information relating to cortical representation of sensory input and the computations acting thereon. While it is difficult to assess, experimentally, the average firing of the entire deep RS population, one can easily do this in the model as one has access and can manipulate every single neuron independently (**Figure 6A**). It is striking that, in the model during SpW, not only do deep RS cells fire more, but there is much greater synchrony, i.e., less dispersion of action potential timing, as compared with delta. In other words, the division of labor between the rhythm generator and pattern generator components of the normal delta rhythm is deranged such that both become facets of a unified rhythm generator – pattern generation, in terms of information held in individual cortico-cortical RS cell spikes, is lost. It is therefore possible that the associated loss of timing information could be related to the cognitive disconnection – the absence – during clinical SpW.

## Conclusion

The examples shown here, of modulator-induced and modulator-modified cortical oscillations and neuronal bursts, are in the spirit of a research program that we have pursued for many years (Traub and Miles, 1991; Traub and Whittington, 2010). This program is favored by several factors, including these:

(1) In hippocampal, neocortical, cerebellar, and other brain slices, it is indeed possible to induce – robustly – network oscillations, either by direct drug application or by tetanic stimulation (which releases glutamate and activates metabotropic glutamate receptors) (Traub et al., 1996; Fisahn et al., 1998; Middleton et al., 2008). Oscillations at a wide range of frequencies can be induced *in vitro*, or even occur spontaneously, from <1 Hz (Cunningham et al., 2006), delta (see above), theta (Gillies et al., 2002; Leznik et al., 2002), alpha (Authors unpublished observations), beta, and gamma (Whittington et al., 1997a,b), up to very fast oscillations or ripples (Maier et al., 2003).
(2) The biological preparations are small enough (thousands of cells) that one can hope to simulate all of the real cells within the computer. Whether this can be done sufficiently accurately is another matter.
(3) As we have discussed, during *in vitro* experimental network oscillations, cells of a given type (say, L5 IB cells) tend to behave similarly during any given oscillation. [But they may not behave identically: for example, during persistent gamma oscillations, different pyramidal cells may fire on the peaks of different gamma waves; or during a synchronized burst, phase differences in burst onset will exist between different pyramidal cells (Traub and Wong, 1982). Indeed, such variability may sometimes be the most interesting observation to be explained given the relationship between it and information content held within spike trains (Fetterhoff et al., 2015). This may turn out to be true in neocortical delta as well.]
(4) The combination of (relatively) small network size and cellular stereotypy reduces the number of “degrees of freedom” in the system, rendering analysis much more tractable than it otherwise would be. However, the actions of modulators – of which there are a great many – again increase the number of degrees of freedom.

Indeed, the whole biological purpose of having modulators is presumably to be able to increase the number of degrees of freedom (in a given circuit), in a controllable way. This allows a given fixed circuit of neurons to be used for a number of distinct purposes (in addition to the obvious ability to control oscillation amplitude and phase) – a principle that has been repeatedly emphasized for invertebrate CPGs (Selverston, 2010; Marder, 2012). The additional complexity neuromodulation brings to even simple circuits is extended further when one considers meta-modulation (Stein, 2009). A biological “payoff” is (presumably) that the brain does not have to be as large as it would otherwise have to be, without modulators – the latter case, an absence of modulators, would presumably require a great deal of redundancy in the circuitry, and hence many more neurons. The difficulty – thinking about it as an engineer might – is how to develop, over the course of evolution, appropriate modulators that lead to useful network behaviors, without leading to pointless or destructive ones (e.g., flat line behavior, or epilepsy); and also, to be able to regulate the modulators themselves. Note as well these complexities: each modulator involves its own synthesis, release, receptors, membrane channels, and/or signaling pathways; and a newly developed/evolved modulator may interact with receptors for the old modulators (c.f. the cross-reactions between ACh and 5HT3a receptors), causing “engineering” confusion.

A final issue concerns how far can the type of research program which we have discussed be pushed. While this is too complex a question to discuss in detail, in a review such as this, it may be appropriate to raise a few questions. First, how much basic experimental information must be acquired about a very large neuronal circuit (say, hundreds of thousands of neurons or more, consisting of many neuronal types), in order to simulate that circuit in a useful way? Next, what sort of experimental paradigms and questions exist, for which a large network model might be useful? The brain does more than oscillate; and even when oscillations are present, each brain region tends to oscillate in its own way [just as every invertebrate CPG has its own properties (Selverston, 2010)]. If the goal of a network simulation is to “reproduce the biology,” what does that mean exactly? There must be a framework for comparing simulations to experimental recordings; in the type of case we have discussed, the framework is – at least in principle – straightforward. But in a more general case, the framework seems ill-defined indeed.

## Author Contributions

All authors listed have made a substantial, direct and intellectual contribution to the work, and approved it for publication.

## Conflict of Interest Statement

The authors declare that the research was conducted in the absence of any commercial or financial relationships that could be construed as a potential conflict of interest.

## Abbreviations

ACh: acetylcholine
AMPA: α-amino-3-hydroxy-5-methyl-4-isoxazolepropionic acid
CPG: central pattern generator
dTC: d-tubocurarine
ECoG: electrocorticography
EEG: electroencephalography
5HT3a: an ionotropic subtype of serotonin (5HT, or 5-hydroxytryptamine) receptor
IB: intrinsic bursting
L5: (neocortical) layer 5
NG: neurogliaform
NMDA: *N*-methyl D-aspartic acid
RS: regular spiking
SOM: somatostatin
SpW: spike-wave
sz: seizure
VIP: vasoactive intestinal peptide
